# Supplementary material for: Nonlinear force dependence on optically bound micro-particle arrays in the evanescent fields of fundamental and higher order microfibre modes
Source: Sci Rep. 2016 Jul 25;6:30131. doi: 10.1038/srep30131 (PMC4958960; doi:10.1038/srep30131)
Supplement: Supplementary Information [file srep30131-s1.pdf]

Nonlinear force dependence on optically bound micro-particle arrays in the evanescent fields of fundamental and higher order microfibre modes

Aili Maimaiti[1,2], Daniela Holzmann[3], Viet Giang Truong[1], Helmut Ritsch[3] & Síle Nic Chormaic*[1]

[1]Light-Matter Interactions Unit, Okinawa Institute of Science and Technology Graduate University, Onna, Okinawa 904-0495, Japan
[2]Physics Department, University College Cork, Cork, Ireland
[3]Institute for Theoretical Physics, University of Innsbruck, Technikerstrasse 25, A-6020, Innsbruck, Austria
*Correspondence to: sile.nicchormaic@oist.jp

Description for the supplementary movies:

Supplementary Movie S1. Propulsion of bounded three polystyrene particles in the evanescent fields of the fundamental mode (FM) of a 2 μm fibre. The power at the fibre waist is 30 mW.

Supplementary Movie S2. Propulsion of bounded four polystyrene particles in the evanescent fields of the fundamental mode (FM) of a 2 μm fibre. The power at the fibre waist is 30 mW.

Supplementary Movie S3. Propulsion of bounded three polystyrene particles in the evanescent fields of the higher order modes (HOMs) of a 2 μm fibre. The power at the fibre waist is 30 mW.

Supplementary Movie S4. Propulsion of bounded four polystyrene particles in the evanescent fields of the higher order modes (HOMs) of a 2 μm fibre. The power at the fibre waist is 30 mW.
